# Pharmacokinetics of micafungin in patients treated with extracorporeal membrane oxygenation: an observational prospective study

**DOI:** 10.5935/0103-507X.20200044

**Published:** 2020

**Authors:** Marta López-Sánchez, Ignacio Moreno-Puigdollers, Maria Isabel Rubio-López, Iratxe Zarragoikoetxea-Jauregui, Rosario Vicente-Guillén, Maria Pilar Argente-Navarro

**Affiliations:** 1 Departamento de Medicina Intensiva, Hospital Universitario Marqués de Valdecilla - Santander, Espanha.; 2 Departamento de Anestesiologia e Reanimação, Hospital Universitario La Fé - Valencia, Espanha.

**Keywords:** Extracorporeal membrane oxygenation, Micafungin/pharmacokinetics, Adult, Oxigenação por membrana extracorpórea, Micafungina/farmacocinética, Adulto

## Abstract

**Objective:**

To determine micafungin plasma levels and pharmacokinetic behavior in patients treated with extracorporeal membrane oxygenation.

**Methods:**

The samples were taken through an access point before and after the membrane in two tertiary hospitals in Spain. The times for the calculation of pharmacokinetic curves were before the administration of the drug and 1, 3, 5, 8, 18 and 24 hours after the beginning of the infusion on days one and four. The area under the curve, drug clearance, volume of distribution and plasma half-life time with a noncompartmental pharmacokinetic data analysis were calculated.

**Results:**

The pharmacokinetics of the values analyzed on the first and fourth day of treatment did not show any concentration difference between the samples taken before the membrane (Cin) and those taken after the membrane (Cout), and the pharmacokinetic behavior was similar with different organ failures. The area under the curve (AUC) before the membrane on day 1 was 62.1 (95%CI 52.8 - 73.4) and the AUC after the membrane on this day was 63.4 (95%CI 52.4 - 76.7), p = 0.625. The AUC before the membrane on day 4 was 102.4 (95%CI 84.7 - 142.8) and the AUC was 100.9 (95%CI 78.2 - 138.8), p = 0.843.

**Conclusion:**

The pharmacokinetic parameters of micafungin were not significantly altered.

## Abstract (pt)

**Objetivo:**

Determinar os níveis plasmáticos e o comportamento farmacocinético da micafungina em pacientes tratados com oxigenação por membrana extracorpórea.

**Métodos:**

As amostras foram colhidas por meio de pontos de acesso antes e depois da membrana, em dois hospitais espanhóis de nível terciário. Os momentos para o cálculo das curvas farmacocinéticas foram antes da administração do fármaco, e 1, 3, 5, 8, 18 e 24 horas após o início da infusão nos dias 1 e 4 de tratamento. Calcularam-se a área sob a curva, a depuração do fármaco, o volume de distribuição e a meia-vida plasmática por meio de análise farmacocinética não compartimental.

**Resultados:**

Os valores farmacocinéticos analisados no primeiro e quarto dias de tratamento não mostram qualquer diferença de concentração entre amostras colhidas antes da membrana e após a membrana, e o comportamento farmacocinético foi similar na vigência de diferentes falências de órgãos. A área sob a curva antes da membrana no dia 1 foi de 62,1 (IC95% 52,8 - 73,4) e a área sob a curva após a membrana nesse mesmo dia foi de 63,4 (IC95% 52,4 - 76,7), com p = 0,625. A área sob a curva antes da membrana no dia 4 foi de 102,4 (IC95% 84,7 - 142,8), enquanto a área sob a curva após a membrana nesse mesmo dia foi de 100,9 (IC95% 78,2 - 138,8), com p = 0,843.

**Conclusão:**

Os parâmetros farmacocinéticos da micafungina não foram alterados significantemente.

## INTRODUCTION

The use of extracorporeal membrane oxygenation (ECMO) has increased in recent years, mainly due to the good results obtained during the influenza A (H1N1) epidemic in 2009^(1-3)^ and the technological breakthroughs made in systems (centrifugal pumps, long-lasting membranes and the biocompatibility of all the components, together with reduced systemic anticoagulation requirements).^(4)^ The system provides two types of support: respiratory (veno-venous - VV - ECMO) and cardiorespiratory (veno-arterial - VA - ECMO). In VV ECMO, a thick venous cannula (femoral or jugular) draws blood from the patient and is driven by a centrifugal pump through a membrane oxygenator. The oxygenated blood is returned to the patient by another femoral or jugular venous cannula. In VA ECMO, blood is returned via an arterial cannula (aortic, subclavian or femoral).^(5)^ It is well known that critically ill patients are subject to important pharmacokinetic (PK) changes that are determined by the presence of an inflammatory response, organ dysfunction, drug interactions, hypoalbuminemia, decreased renal clearance and the use of support treatments such as continuous renal replacement therapies and ECMO.^(6,7)^ Generally, the volume of distribution (Vd) increases and drug clearance (CL) and elimination decrease during ECMO, but in patients with systemic inflammatory response syndrome/sepsis, or because of drug sequestration by the circuit, clearance is increased.^(8)^ The optimization of antimicrobial doses by means of dose titration and the determination of plasma levels according to PK/pharmacodynamic changes may improve the survival and clinical evolution of critically ill patients.^(6)^ The initiation of ECMO yields a series of important PK changes that are described below. Hemodilution, the priming of tubing, drug sequestration, organ failure and the hydrophilicity of the drug increase Vd.^(7,8)^ Moreover, drug sequestration takes place at the oxygenation membrane level and in the rest of the system, depending on lipophilicity, ionization, plasma protein binding and the drug’s molecular size,^(7)^ with greater sequestration of lipophilic and highly protein-bound drugs.^(7,9)^ Decreased plasma protein binding increases the unbound drug concentration, resulting in an increased Vd, especially for highly protein-bound drugs.^(7)^ The lower renal clearance of drugs in the presence of kidney failure and the concomitant use of continuous renal replacement therapies can increase CL and are two factors that alter the elimination of drugs in patients on ECMO. Drug metabolism can be altered in ECMO patients because the acute reduction in hepatic blood flow and the alteration of hepatic enzyme function in ECMO reduces clearance of hepatically cleared medications.^(7-8)^.^( )^The majority of PK studies in ECMO have been performed in newborns, and sedatives, analgesics and antimicrobials are the best-studied drugs. In adults, few studies have been published with regard to antifungals in patients treated with ECMO.^(10,11)^ Voriconazole (a lipophilic drug) levels are reduced, and the monitoring of its plasma levels is recommended.^(10,11)^ The levels are variable in the case of caspofungin.^(10,11)^ A recent PK study with micafungin in a pediatric population demonstrated a greater Vd and CL in ECMO patients versus non-ECMO patients.^(12)^

The objective of this research was to study the PK of micafungin in critically ill patients treated with ECMO. Knowledge of drug behavior in these patients is necessary for correct management and dosing of micafungin.

## METHODS

We conducted an observational, prospective, noninterventional and nondrug-linked study of the PKs and safety of micafungin in adult patients treated with ECMO in two Spanish hospitals: the intensive care unit (ICU) of the *Hospital Universitario Marqués de Valdecilla,* Santander, and the Anaesthesiology and Reanimation Department of the *Hospital Universitario La Fe*, Valencia, Spain. Prophylactic micafungin was administered in all patients. The study period was from January 2015 until April 2016. The PK analysis was performed in the *Hospital del Mar*, Barcelona, Spain. The trial was authorized by the corresponding Ethics Committees (CEIC Approval Number 17/2014), and Informed Consent of all the patients or their legal representatives was obtained.

Inclusion criteria were adult patients (18 years or older) treated with ECMO who received or were receiving treatment or prophylaxis with micafungin due to the confirmed or suspected presence of fungal infection were included in the study. Exclusion criteria were patients participating in other clinical trial during the study period and patients scheduled to be transferred to another hospital department or another hospital within 24 hours following the date of their inclusion in the study were excluded from the study.

Study variables: demographic data collected included age, weight, sex, height, body mass index, concomitant diseases, Acute Physiology and Chronic Health Evaluation (APACHE) II score, Simplified Acute Physiology Score (SAPS) II, *Candida* score, days of stay in the ICU), diagnosis on admission to the ICU, daily analytical data (liver function, renal function, total protein level, albumin and other liver profile parameters, full blood count and clotting parameters), diuresis, and daily water balance. ECMO data included the type of support (VV or VA), cannula position, priming volume, number of membrane replacements and type thereof, and ECMO flow at the time the samples were taken.

Acute kidney injury was defined as an increase in serum creatinine by 26µmol/L over baseline in 48 hours, an increase in creatinine by 1.5 times the baseline value, or urinary output < 0.5mL/kg/hour for 6 hours.

Acute hepatic insufficiency was defined by transaminase and bilirubin elevation three times the baseline value together with an International Normalized Ratio (INR) > 1.5 or prothrombin activity < 50%.

The adverse effects of micafungin were recorded, as well as any associated with the use of hepatotoxic drugs in combination with micafungin.

Micafungin was given at a dose of 100mg diluted in 100ml of 0.9% saline in a 60-minute infusion always protected from light to prevent degradation. Five mL blood samples were taken, protected from light, on days 1 and 4 of the treatment with micafungin. The samples were taken through an access point before and after the oxygenation membrane (input and output, respectively). The extraction times for the calculation of complete PK curves were as follows: before the administration of the drug (trough or zero time); immediately after intravenous infusion (1-hour peak or end-of-infusion peak); and 3, 5, 8, 18 and 24 hours after the beginning of the infusion. Samples were centrifuged at a speed of 1,000g for ten minutes at 4ºC. The supernatant plasma was aliquoted into volumes of 150µl that were frozen at -80ºC until they were to be analyzed. The measurement of the total micafungin concentration (free plus protein-bound fractions) was performed by means of a high-performance liquid chromatography technique. It is a linear analytical technique (coefficient of linearity - CL > 0.99) throughout the range of concentrations studied, with accuracy values between 85% and 115% and precision (coefficient of variation < 20%) for intraday and interday variability, respectively, and with a limit of quantitation of 0.5mg/L. The plasma concentration over time curve was generated with micafungin concentrations at the established time frames.

The following PK parameters were calculated: area under the curve (AUC), CL, Vd and plasma half-life time (t/2) with a noncompartmental PK data analysis using a nonparametric method: the Mann-Whitney test and the Wilcoxon signed-rank test (paired test, in consecutive days). For precision analysis, confidence intervals were calculated by bootstrapping with 1,000 repetitions using the method “percentile bootstrap adjusted” (BCa).

The calculation of the differences in the concentration of micafungin at the input (Cin) and output (Cout) of the oxygenator membrane allowed us to calculate the oxygenator’s degree of extraction. It should be remembered that there may be losses of micafungin in tubes and cannula and in the centrifugal pump.

Only continuous epidemiological and clinical data are presented as the mean and standard deviation (SD), and categorical data are presented as absolute numbers because of the small sample size. The rest of the parameters, including Cmin and Cmax, are presented as medians.

The comparison of all parameters between “in” and “out” was always statistically not significant, with p > 0.5 in all cases, on day 1 and on day 4.

When we compared “in” and “out”, each patient served as their own control, and we used the Wilcoxon test. This is the same when we compared day 1 and day 4. When we compared different groups, for example, men and women, we used a test for independent samples (the Mann-Whitney test).

PKSolver software was used to estimate the noncompartmental model, and the R program was used to perform statistical analyses.

## RESULTS

Of the 12 patients recruited between 18 March 2015 and 18 January 2016 (10 months), ten from *Hospital Universitario La Fe* and two from *Hospital Universitario Marqués de Valdecilla.* Eight were men (66.7%), and the mean age of the whole cohort was 54 (SD of 13) years (Table 1).

**Table 1:** Demographic and clinical data

	N = 12
Age (years)	54 ± 13
Sex	8/12
Weight (kg)	71 ± 14
Diagnosis	
Postcardiotomy cardiogenic shock	9/12
Right ventricular failure in lung transplant	2/12
Respiratory failure bridge to lung transplant	1/12
APACHE II	24 ± 6
AKI	4/12
Hepatic failure	5/12
ECMO support	
Veno-arterial	10/12
Veno-venous	2/12
ECMO circuit membrane	
HLS	11/12
PLS	1/12
Plasma proteins (g/L)	46 ± 11
Total bilirubin (µmol/L)	30.8 ± 15.4

APACHE - Acute Physiology and Chronic Health Evaluation; AKI - acute kidney injury; ECMO - extracorporeal membrane oxygenation. Results expressed as mean ± standard deviation or n/total n.

The indication for VA ECMO was postcardiotomy cardiogenic shock or right ventricular failure (n = 9) and intraoperative respiratory support for a patient undergoing lung transplant (n = 1). The two remaining patients required VV ECMO (16.7%) for acute respiratory distress syndrome or respiratory failure as a bridge to lung transplantation. Cannulation was performed in all patients by means of a peripheral femoral line. The ECMO MAQUET Cardiohelp® (Rastatt, Germany) with an HLS membrane was used in the majority of the patients (91.7%), except in one patient (8.3%), in whom the PLS membrane, also by MAQUET, was used. Thirty-three percent of the patients presented some degree of acute kidney injury according to the Kidney Disease: Improving Global Outcomes (KDIGO) criteria, although no patient required renal replacement therapy. Using the definitions of transaminase and bilirubin level elevations three times from the baseline levels together with an INR of greater than 1.5 or prothrombin activity less than 50%, acute liver insufficiency was observed in 41.7% of the patients.

**Figure 1:**
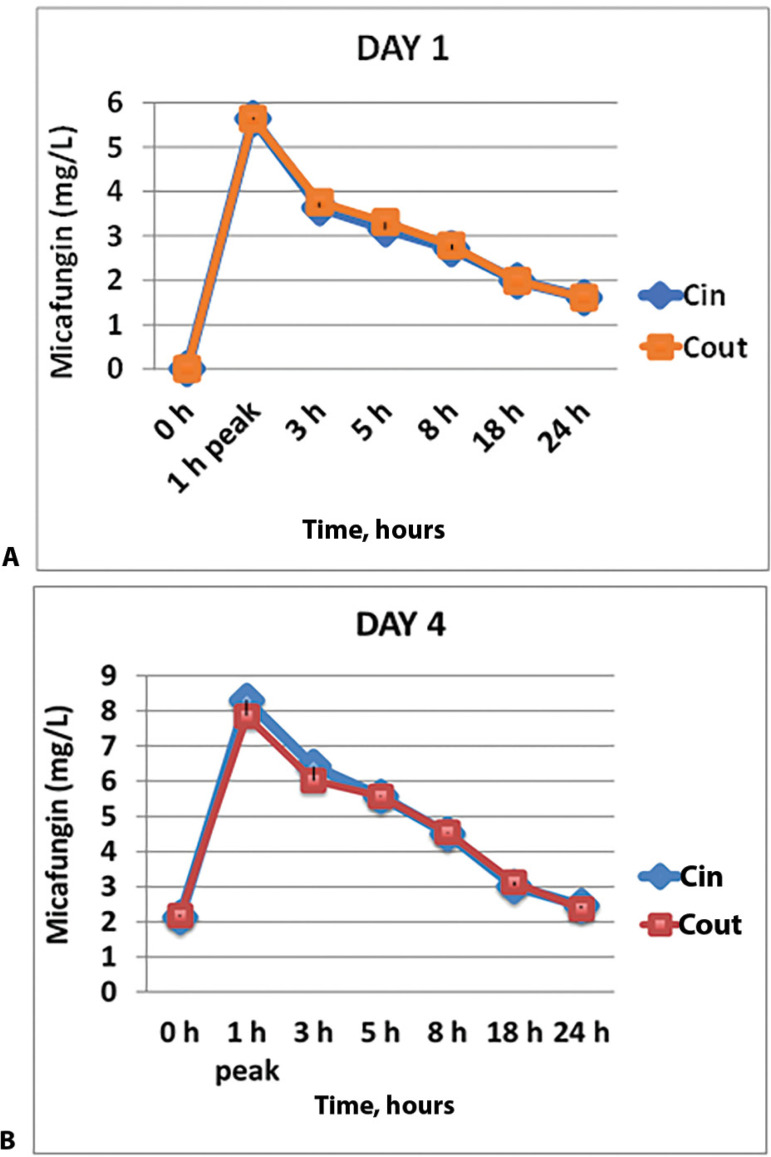
Micafungin concentrations determined in samples over 24 hours from the venous pre-extracorporeal membrane oxygenation port (Cin) and the venous postextracorporeal membrane oxygenation port (Cout) on day 1 (A) and day 4 (B).

Samples were taken from ten patients on the first day of treatment with micafungin and from eight patients on the fourth day of therapy. Of the 12 patients who had samples collected, six had samples taken on both the first and fourth days of treatment. The PKs of micafungin on the first day of treatment with micafungin did not show any concentration difference between the samples taken before the membrane (C in) and those taken after the membrane (C out), as shown in figure 1A. The AUCin on day one was 62.1mg.h/L (95% confidence interval - 95%CI 52.8 - 73.4), and the AUCout on day one was 63.4mg.h/L (95%CI 52.4 - 76.7), p = 0.625. The other PK data for day one (Cmax, Cmin, Vd and Cl) are shown in table 2 (no significant differences). The PKs of micafungin on the fourth day of treatment with micafungin did not show any concentration difference between the samples taken before the membrane (Cin) and those after the membrane (Cout), as shown in figure 1B. The AUC in on day 4 was 102.4mg.h/L (95%CI 84.7 - 142.8), and the AUCout on day four was 100.9mg.h/L (95%CI 78.2 - 138.8), p = 0.843. The other PK data for day four (Cmax, Cmin, Vd, Cl) are shown in table 2 (no significant differences). No significant differences were found comparing the AUC out of days 1 and 4 of treatment in the patients with acute kidney or liver injury compared to those without these complications (Table 3). No candidemia was observed in patients on ECMO who received prophylactic micafungin.

**Table 2:** Pharmacokinetic parameters on days 1 and 4

	AUC in (mg.h/L)	AUC out (mg.h/L)	Vd in (L)	Vd out (L)	Cmax in (mg/L)	Cmax out (mg/L)	Cmin in (mg/L)	Cmin out (mg/L)	CL in (L/h)	CL out (L/h)
**Day 1**	54.9	54.1	28.6	27.2	5.85	4.95	1.41	1.41	1.07	0.99
**Day 4**	88.8	81.0	16.1	18.1	12.20	6.75	1.80	1.75	0.71	0.77

AUC - area under the curve; Vd - volume of distribution; Cmax -concentracion maximun; Cmin -concentracion minimun; CL - coefficient of linearity. Concentracion maximun and concentracion minimun are presented as medians. The area under the curve, coefficient of linearity and volume of distribution were analyzed by the Wilcoxon signed-rank test and the Mann-Whitney test.

**Table 3:** Pharmacokinetic parameters with acute kidney injury or hepatic failure on days 1 and 4

	No AKI AUC_out_	AKI AUC_out_	No hepatic failure AUC_out_	Hepatic failure AUC_out_
**Day 1**	61.9	69.3 p = 0.71	66.1	61.6 p = 0.91
**Day 4**	95.1	106.7 p = 0.88	116.6	85.2 p = 0.68

AKI - acute kidney injury; ASC - area under the curve. The area under the curve was analyzed by the Wilcoxon signed-rank test and the Mann-Whitney test.

## DISCUSSION

Patients requiring ECMO have unstable physiology, with multiorgan failure either as a result of the primary disease leading to the requirement for ECMO or as a result of when the critical state is complicated by nosocomial infections.^(13)^ Additionally, ECMO use can be an important contributor to ECMO-related systemic inflammation.^(14)^ The presence of acquired infections in ECMO patients has a 50% associated mortality rate.^(15)^ As such, empirical or even prophylactic antimicrobial therapy is commonly prescribed, assuming that such therapy would achieve the same targeted efficacy as the standard recommended doses for patients not treated with ECMO. There are very few studies on the use of antifungals in patients during ECMO treatment. To the best of our knowledge, this is the first PK analysis of an echinocandin in a series of adult patients treated with ECMO. When fluconazole is prescribed for children treated with ECMO, the dose needs to be increased,^(16)^ and levels of voriconazole monitored.^(10)^ Currently, data on anidulafungin^(17,18)^ and caspofungin^(10,11)^ are very limited. Plasma levels, AUC and the other PK data of micafungin obtained in the study are consistent with those observed in other studies in critically ill patients^(19,20)^ or those undergoing extracorporeal renal replacement techniques.^(21)^ The absence of the absorption of micafungin by the ECMO membrane and circuit has been demonstrated, thus ruling out the need to increase drug doses in patients on this type of mechanical assistance, particularly during the maintenance phase of drug therapy. These findings were also observed previously by different authors, albeit in isolated cases^(22)^ or in children,^(12)^ who present a greater Vd and clearance.

A study comparing micafungin pharmacokinetics in a control group of non-ECMO patients to those of ECMO patients found a 23% reduction in the AUC in the ECMO group, but hemodialysis was used in 4 patients.^(23)^ None of our patients needed hemofiltration. Adsorption by the hemoﬁlter appears to be the most likely explanation when this is in line with an ECMO system. In an “ex vivo” study, the recovery of micafungin was 91% when the in-line hemofilter was removed.

The plasma concentrations of micafungin in patients with VV ECMO appeared similar to those in patients treated with VA ECMO, suggesting that the presence of recirculation in VV ECMO (a portion of the oxygenated blood returned to the venous system is immediately taken back into the ECMO circuit via the drainage lumen of the cannula; this phenomenon would increase the time the drug spends within the ECMO circuit) may not affect its PKs.^(24,25)^ We did not observe any significant differences in the PKs of micafungin between patients with and without acute kidney or liver injury, similar to the results of some other reports.^(19,26)^ This would suggest that we do not need to reduce doses of micafungin for these patients. That being said, concentrations of albumin in subjects with severe hepatic dysfunction are lower, and this may increase the micafungin-free fraction even though there is a lower total plasma concentration with a reduction in AUC. According to other authors, this is not considered to be clinically relevant, and dose adjustments are not recommended for patients with moderate or severe hepatic dysfunction.^(27)^ The AUC after a daily dose of 100mg micafungin in healthy adults was 132.6mg.h/L, and in ICU patients, it was 78.6mg.h/L.^(19)^ Intensive care unit patients (without ECMO) are subject to severely altered PK characteristics, including variations in the Vd and CL, compared to noncritically ill patients. Whether a lower AUC would have significant clinical consequences is subject to debate. If we aim for an AUC closer to those achieved in healthy adults for patients treated with ECMO, a loading dose of 200mg and subsequent maintenance of 150mg/day will be necessary to obtain adequate plasma levels, especially if patients have hepatic dysfunction when a lower AUC is expected.^(19)^ The micafungin concentrations measured in the maintenance phase of drug treatment were higher than the MIC90 published for *Candida albicans* (0.03µg/mL).^(27,28)^ The area under the plasma drug concentration-versus-time curve/minimum inhibitory concentration (MIC) values was 3,413 for *C. albicans*. An AUC/MIC > 3,000 (clinical efficacy for nonparapsilosis *Candida* species)^(26,27)^ was achieved in the maintenance phase using cut-off points < 0.034mg/L.

Our data suggest that adequate plasma levels of micafungin can be achieved in critically ill patients with highly complex cases, with and without acute kidney and liver injury, who require ECMO without hemofiltration. However, this study’s generalizability is limited by its small sample size and a lack of data relating the PK information to any patient-centered outcomes.

## CONCLUSION

In our series of patients treated with extracorporeal membrane oxygenation, the pharmacokinetic parameters of micafungin were not significantly altered, including those in patients with mild to moderate acute kidney or liver injury.
